# Astaxanthin Supplementation Delays Physical Exhaustion and Prevents Redox Imbalances in Plasma and Soleus Muscles of Wistar Rats

**DOI:** 10.3390/nu6125819

**Published:** 2014-12-12

**Authors:** Tatiana G. Polotow, Cristina V. Vardaris, Andrea R. Mihaliuc, Marina S. Gonçalves, Benedito Pereira, Douglas Ganini, Marcelo P. Barros

**Affiliations:** 1 Institute of Physical Activity and Sports Sciences (ICAFE), Cruzeiro do Sul University, 01506-000 Sao Paulo, Brazil; E-Mails: tatianapolotow@hotmail.com (T.G.P.); crisvardaris@gmail.com (C.V.V.); andreamihaliuc@hotmail.com (A.R.M.); masg_je@yahoo.com.br (M.S.G.); douganini@uol.com.br (D.G.); 2 School of Physical Education and Sports (EEFE), University of Sao Paulo (USP), 05508-900 Sao Paulo, Brazil; E-Mail: benepe@usp.br; 3 Free Radical Metabolism Group, Laboratory of Toxicology and Pharmacology, National Institute of Environmental Health Sciences, NIEHS, Research Triangle Park, Durham, NC 27709, USA

**Keywords:** carotenoid, exercise, iron, uric acid, oxidative stress, mitochondria

## Abstract

Astaxanthin (ASTA) is a pinkish-orange carotenoid commonly found in marine organisms, especially salmon. ASTA is a powerful antioxidant and suggested to provide benefits for human health, including the inhibition of LDL oxidation, UV-photoprotection, and prophylaxis of bacterial stomach ulcers. Exercise is associated to overproduction of free radicals in muscles and plasma, with pivotal participation of iron ions and glutathione (GSH). Thus, ASTA was studied here as an auxiliary supplement to improve antioxidant defenses in soleus muscles and plasma against oxidative damage induced by exhaustive exercise. Long-term 1 mg ASTA/kg body weight (BW) supplementation in Wistar rats (for 45 days) significantly delayed time to exhaustion by 29% in a swimming test. ASTA supplementation increased scavenging/iron-chelating capacities (TEAC/FRAP) and limited exercise-induced iron overload and its related pro-oxidant effects in plasma of exercising animals. On the other hand, ASTA induced significant mitochondrial Mn-dependent superoxide dismutase and cytosolic glutathione peroxidase antioxidant responses in soleus muscles that, in turn, increased GSH content during exercise, limited oxidative stress, and delayed exhaustion. We also provided significant discussion about a putative “mitochondrial-targeted” action of ASTA based on previous publications and on the positive results found in the highly mitochondrial populated (oxidative-type) soleus muscles here.

## 1. Introduction

**Figure 1:**
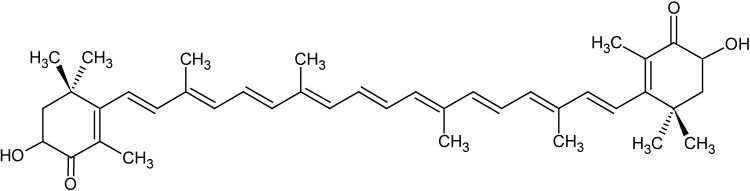
Chemical structure of the algal carotenoid astaxanthin (ASTA).

The algal carotenoid astaxanthin (ASTA; Figure 1) is a pinkish-orange pigment naturally found in many marine organisms, such as crustaceans and fishes (especially salmon). ASTA is a powerful antioxidant, both *in vitro* and *in vivo*, especially against peroxyl (ROO•) and alkoxyl radicals (RO•), the main propagating radicals of lipid peroxidation in membranes [1]. Recently, ASTA was shown to act, even at nanomolar concentrations, as an important factor to sustain membrane potential (∆Ψ), high rates of respiration, and the redox state of isolated mitochondria exposed to oxidative conditions [2]. Based on its antioxidant properties, ASTA affords UV-photoprotection to skin/eyes, enhances immune responses, protects against gastric ulcer induced by *Helicobacter pylori*, and displays cardioprotective effects, among many other benefits to human health [3].

Skeletal muscles produce reactive oxygen species (ROS) during contractile activity and even at rest [4]. However, intracellular ROS sources other than mitochondria might be significantly involved in ROS production during exercise, depending on the prevalent energy-demanding activity (aerobic/endurance or anaerobic/resistance), frequency, intensity, and duration [5]. Aerobic exercise is associated with a substantial increase in O_2_ uptake by contractile muscles (up to 80-fold higher in oxygen volume), activation of oxidative mitochondrial metabolism and, thus, increased mitochondrial production of ROS with minor contributions from other cellular sources [6]. Exercise-related oxidative stress increases by the release of iron ions in extracellular fluids and plasma during/after exercise, although by still unrevealed mechanisms [7]. Several plasma biomarkers of oxidative stress have been investigated in animal models and humans for an accurate evaluation of the relationship between physical effort/performance and free radical-based injuries and diseases [8].

Redox imbalances during muscular contractions can significantly contribute to the reduction of the contractility force, precocious fatigue, and higher susceptibility to muscular injuries [9]. Therefore, antioxidant supplementation could hypothetically improve performance as well as circumvent muscle disorders, depending on the biochemical and pharmacological properties of the antioxidant compound [4]. Therefore, this work aimed to evaluate the effects of the long-term supplementation with the marine carotenoid ASTA on exhaustion and redox biomarkers in plasma and the soleus muscle (mostly involved in endurance/aerobic exercise) of Wistar rats submitted to forced-swimming activity.

## 2. Experimental Section

### 2.1. Chemicals

All chemicals were of analytical grade and purchased from Sigma-Aldrich (St. Louis, MO, USA), except those used for usual solutions, including specific buffers, which were purchased from Labsynth (Diadema, SP, Brazil). Chromatography-quality solvents were obtained from Merck (Düsseldorf, Germany), and all stock solutions and buffers were prepared with Milli-Q purified water (Millipore, Billerica, MA, USA).

### 2.2. Astaxanthin Source

Natural ASTA supplements (AstaREAL A1010) were obtained as a donation from the Swedish company BioReal AB (Gustavsberg, Sweden), a subsidiary of BioReal Inc. (Kihei, HI, USA) and part of the pharmaceutical Group Fuji Chemical Industry CO. AstaREAL A1010 is an astaxanthin-rich natural microalgal product, consisting of crushed and spray-dried aplanospores of the green microalga *Haematococcus pluvialis*. AstaREAL A1010 contains 42% of crude fat, 10% of crude protein, 40% of carbohydrates, and 4% of ashes. Regarding carotenoid composition, AstaREAL A1010 contains 5.0%–5.6% of pure ASTA (3.9% as monoesters, 0.9% as diesters, and 0.1% in free form), 0.02% of lutein/zeaxanthin, 0.02% of adonirubin, 0.02% of cantaxanthin, 0.02% of β-carotene, and 0.1% of others. Although other antioxidants such as ascorbate and tocopherols are also residually present in AstaREAL A1010, their contribution in the total antioxidant capacity of gavage solutions is minor if compared to the prevalent ASTA component. We analyzed ASTA content in AstaREAL biomass by extracting carotenoids in acetone and measuring absorbance at 473 nm (compared to a standard curve) before supplementation and observed similar results as reported by BioREAL AB: (4.1 ± 1.7) mg ASTA/100 mg AstaREAL biomass [10,11]. The ASTA stock solution (approximately 20 mg AstaREAL/mL, which contains 0.82 mg ASTA/mL) was prepared in mineral oil, by grinding AstaREAL biomass in a mortar and dissolving it by mixing manually, with the help of an ultrasound bath (at 25 °C to avoid carotenoid oxidation) or by sonication, when necessary. Proportional to animal weights, different volumes of ASTA stock solution were administered to animals by gavage to reach a daily ingestion of 1 mg ASTA/kg BW (5-times/week).

### 2.3. Animals and Supplementation Protocols

Adult Wistar male rats, weighing approximately 150 g at the beginning of the study were provided by the Department of Psychobiology, Universidade Federal de São Paulo (UNIFESP), São Paulo, Brazil. All animals were housed in Plexiglas cages (5 rats/cage), under standard laboratory conditions: 12 h light/dark cycle; lights on at 7:00 a.m., (23 ± 1) °C, and *ad libitum* access to water and Purina rat chow (20% of protein, as 98% casein, 7% of cellulose, 74% of carbohydrates, and 4% of fat). Animals were handled in accordance with guidelines for care and use of laboratory animal resources. The Ethics Committee for experimental animals from Universidade Cruzeiro do Sul approved all the experimental protocols described here, including supplementation and exercise protocols (CE/UCS-194/2011). The animals were treated 5 days/week with mineral oil (control) or ASTA solution (dissolved in mineral oil), for a total of 45 days. A maximum volume of 0.5 mL was established for the gavage treatment in order to avoid regurgitation or stomach discomfort of the animals.

### 2.4. Exercise Protocol

After 45 days of supplementation, the animals were randomly split into two groups: rested (C and ASTA, depending on supplementation), or exercised to exhaustion (C-EX and ASTA-EX). A larger number of animals was used to collect swimming performance data (*n* = 11 for control group, and *n* = 14, for ASTA group), than for biochemical parameters (*n* = 6, for all experimental groups). All animals were weighted and additional weights (5% of BW) were fixed on the tails of C-EX and ASTA-EX animals to force the swimming activity [12]. C-EX and ASTA-EX animals were individually placed in PVC-compartment apparatus, temperature regulated (31 ± 1 °C), and led to swim until exhaustion. Exhaustion was defined as the condition when the animal remained submersed and unresponsive for approximately 3–5 s [13]. After 15 min of the exhaustive test, 24 animals (*n* = 6 per group: C, C-EX, ASTA, and ASTA-EX) were killed by decapitation and total blood content was collected in EDTA-containing Vacutainer^®^ flasks for plasma isolation (by centrifugation for 5 min, at 4× *g*, RT). Soleus muscles are rich in slow-twitch type I fibers, provided with higher oxidative metabolism and, therefore, of major contribution on the swimming exercise taken until exhaustion [14]. Soleus muscles of both legs were excised, wrapped, immediately stored in liquid nitrogen, and then in a −80 °C freezer for further biochemical analyses.

### 2.5. Glucose, Triacylglycerol and Cholesterol Determinations in Plasma

Glucose, triacylglycerol (TAG), and cholesterol levels in plasma were quantified by commercial test kits obtained from Bioclin-Quibasa Química Básica (Belo Horizonte, MG, Brazil). All assays were based on the peroxidase-catalyzed oxidation of 4-aminoantipyrine by H_2_O_2_ to generate a pinkish chromophore detected at 520 nm.

### 2.6. Plasma Biomarkers of Oxidative Stress

#### 2.6.1. Trolox-Equivalent Antioxidant Capacity Assay (TEAC)

The TEAC assay quantifies the scavenging capacity of plasma antioxidants by measuring the kinetic absorbance decay (at 734 nm) of the relatively stable radical of 2,2′-azinobis(3-ethylbenzothiazoline-6-sulfonate), ABTS•-, in 150 mM NaCl, 100 mM phosphate buffer solution, pH 7.4. Plasma samples were compared to different concentrations of 6-hydroxy-2,5,7,8-tetramethylchroman-2-carboxylic acid (Trolox, a water-soluble derivative of α-tocopherol) and, thus, expressed as Trolox equivalents/mL [15].

#### 2.6.2. Ferric-Reducing Activity of Plasma (FRAP)

The ferric-reducing activity of plasma (FRAP) quantifies metal ligands in plasma that form redox inactive complexes [Fe(L)]*^n^*^+^ that, thereby, minimize the catalysis of deleterious Fenton-type reactions and other radical processes [16,17]. The FRAP assay was here performed by replacing the Fe^2+^-chelating agent 2,4,6-tripyridyl-S-triazine (TPTZ) by its analog 2,3-bis(2-pyridyl)-pyrazine (DPP). Briefly, 10–20 µL of samples were mixed with the FRAP reactant solution containing 10 mM DPP (from a stock solution in 40 mM HCl) and 20 mM FeCl_3_ in 0.30 M acetate buffer (pH 3.6). Absorbance at 593 nm was recorded for 4 min in a 96-well microplate reader (SpectraMax M5; Molecular Devices, Sunnyvale, CA, USA) to determine the rate of Fe^2+^-DPP complex formation as compared to a standard curve.

#### 2.6.3. Iron/Heme-Iron Determination in Plasma

Total iron concentration in plasma was assayed using a commercial biochemical kit from Doles-Bioquímica Clínica (Goiania, GO, Brazil) based on the formation of the Fe^2+^:ferrozine complex (at 560 nm) by the reducing mixture of 0.36 M hydroxylamine chloride, 0.10 M glycine, 14 mM thiosemicarbazide, and 0.50 mM octylphenoxypolyetoxyethanol, at pH 2.2. Total heme-iron content in plasma (from hemoglobin, myoglobin and other heme proteins) was assayed based on the heme-iron oxidation by the ferricyanide anion contained in a solution of 0.10 M KH_2_PO_4_, 60 mM K_3_[Fe(CN)_6_], 77 mM KCN, and 82 mM Triton X-100. Heme-iron cyanide was stoichiometrically detected at 540 nm, using hemoglobin as a standard curve [18].

#### 2.6.4. Uric Acid Determination

Plasma uric acid (UA) content was assayed using a commercial biochemical kit from Doles-Bioquímica Clínica (Goiania, GO, Brazil). The method is based on the indirect measure of H_2_O_2_ formed from the oxidation of UA to allantoin by uricase. Then, H_2_O_2_ reacts with p-hydroxybenzoate and 4-aminoantipyrine (catalyzed by peroxidase) to form a pinkish chromophore detected at λ = 505 nm.

### 2.7. Preparation of Soleus Homogenates

Soleus muscles were homogenized in a mechanical tissue grinder, for 2 min, under ice-water bath in order to avoid enzyme degradation. The extraction buffer used in sample preparation for measuring products of protein (carbonyl and thiol groups) and lipid oxidation (thiobarbituric acid-reactive substances; TBARS assay) was the same as for superoxide dismutase (SOD), xanthine oxidase (XO), and catalase (CAT) activity analyses: 50 mM phosphate buffer, pH 7.4. Glutathione peroxidase (GPX) and glutathione reductase (GR) determinations were performed in 0.1 M phosphate buffer, pH 7.4, in the presence of 2 µg/mL leupeptin, and 100 µM phenylmethanesulfonylfluoride (PMSF), a classic protease inhibitor. Finally, reduced and oxidized glutathione determinations (GSH and GSSG, respectively) were performed by electrochemical detection in a liquid chromatography system (EC-HPLC) and samples were homogenized in 0.1 phosphate buffer, pH 7.2. After homogenization, all preparations were subsequently centrifuged (4× *g*, 10 min, 4 °C), debris was discarded, and the supernatant was kept on ice for further analysis.

### 2.8. Antioxidant Enzyme Determination in Muscles

#### 2.8.1. Superoxide Dismutase Assay (SOD)

The activity of SOD was measured as follows: the reaction system included 50 mM sodium phosphate buffer, pH 7.4, 0.1 mM EDTA, 50 µM nitrobluetetrazolium (NBT), 78 µM NADH, and 3.3 µM phenazine methosulphate (PMS) [19]. In order to discriminate different SOD isoforms, assays were repeated in the presence of 3 µM KCN in order to inhibit the cytosolic CuZnSOD isoform and, thus, detect mitochondrial MnSOD separately. Absorbance at 550 nm was monitored for over 2 min and kinetic rates were calculated. One SOD unit is defined as the enzyme concentration required for 50% inhibition of NBT reduction at 25 °C.

#### 2.8.2. Catalase Assay (CAT)

The decomposition of H_2_O_2_ was directly followed for 5 min (absorbance decay at 240 nm), using a molar extinction of ε_240_ = (0.0394 ± 0.0002) L·mM^−1^·cm^−1^ [20]. One CAT unit is defined as the enzyme concentration required for the decomposition of 1 µmol of H_2_O_2_ per min at 25 °C.

#### 2.8.3. Glutathione-Dependent Enzymes Assay

The activity of glutathione peroxidase (GPX) was measured in 0.1 M phosphate buffer, pH 7.4, in the presence of 2.5 U/mL glutathione reductase (GR), 10 mM reduced glutathione (GSH), 250 µM sodium azide (a CAT inhibitor), and 1.2 mM NADPH [21]. The reaction was triggered by 4.8 mM *tert*-butyl hydroperoxide (a GPX substrate). Glutathione reductase (GR) activity was measured by a direct reaction between 3.6 mM NADPH and 10 mM oxidized glutathione (GSSG). In both analyses, the rates of absorbance increase at 340 nm (NADPH oxidation in 0.2 M phosphate buffer, pH 7.4) were recorded for 5 min, at 25 °C, in a SpectraMax 190 microplate reader (Molecular Devices, Sunnyvale, CA, USA).

### 2.9. Xanthine Oxidase Activity in Muscles

The activity of XO was measured in soleus homogenates by calculating the rates of the absorbance increase at 290 nm (formation of uric acid; ε_290nm_ = 12.2 mM^−1^·cm^−1^) in a reaction mix with 33 mM potassium phosphate buffer, pH 7.5, and 0.050 mM xanthine. One unit of XO will convert 1.0 µmol of xanthine to uric acid per minute at 25 °C and pH 7.5 [22].

### 2.10. Biomarkers of Oxidative Modifications in Proteins and Lipids

Carbonyl and thiol groups in total protein fraction of soleus homogenates were measured as indexes of amino acid oxidation. Interfering nucleic acids were removed from samples by a pre-treatment with 10% streptomycin. After nucleic acid clearance, protein fractions were isolated in homogenate by precipitation with 20% trichloroacetic acid solution in ice, followed by the subsequent processes: (i) washing once with 0.3 M HClO_4_, 5 mM EDTA, and 0.06% 2,2′-bipyridine solution; (ii) washing twice with the mixture 1:1 ethyl acetate:ethanol (v/v); and (iii) removal of residual organic solvent in vacuum for, at least, 30 min (SpeedVac apparatus). Then, protein pellets were fully dissolved in 6 M guanidine hydrochloride. Carbonyl groups were detected by a stoichiometric reaction with 10 mM dinitrophenylhydrazine (DNPH) in 0.25 M HCl (blanks lack DNPH). Absorbance was recorded at 380 nm, and the carbonyl group concentration was calculated using an extinction coefficient of ε = 2.2 × 10^4^ M^−1^·cm^−1^ [23]. From the same isolated protein fraction of soleus homogenates, thiol groups were measured based on the reaction of available -SH groups in sample proteins with 2 mM 5,5′-dithiobis(2-nitrobenzoic acid) in 50 mM phosphate buffer, pH 7.0. Absorbance was recorded at 412 nm, using reduced glutathione (GSH) as a standard, and *N*-ethylmaleimide as negative control [24].

The assay of lipid peroxidation was performed by measuring the concentration of thiobarbituric acid-reactive substances (TBARS) in muscle samples. Butylated hydroxytoluene (4% BHT, in ethanol) was added to stop progressing oxidation reactions in 300 µL diluted samples. Pinkish adducts of TBA with lipid aldehydes (products of lipid oxidation) were quantified at 535 nm after reaction of samples with 0.375% thiobarbituric acid in 0.25 M HCl and 1% Triton X-100 (15 min, at 100 °C). Malondialdehyde equivalents (nmol MDAeq./mg protein) were calculated with blanks lacking TBA, and using 1,1,2,2-tetroxyethylpropane (TEP) as standard [25].

### 2.11. Liquid Chromatography Analyses of Glutathione (HPLC)

Reduced and oxidized glutathione (GSH and GSSG, respectively) in 20 μL samples of soleus muscles were simultaneously separated and quantified by liquid chromatography technique (HPLC) in a Shimadzu SCL 10AT-VP system, set for an isocratic flow (1.0 mL·min^−1^) of 50 mM potassium phosphate buffer, pH 7.2, with 50 μM octanesulfonic acid, and 2% acetonitrile, as mobile phase [26,27]. Pre- and running chromatography columns were purchased from Phenomenex^®^: 250 × 4.6 mm, 5 μm mesh, reverse phase LUNA RP-18 column was used. Electrochemical detection (ESA^®^ Coulochem III, plus ESA^®^ 5020, as guide cell) was used for quantification of both GSH and GSSG. Electric potentials of coulometric cells (ESA^®^ 5010) were set for: E1 = 650 mV, and E2 = 850 mV. Chromatography data were directly analyzed by the equipment software (Shimadzu^®^ Class VP 6.12 SP2).

### 2.12. Statistical Analysis

Statistical analysis was conducted using the Origin 6.1 software (v6.1052/B232; OriginLab Corporation, Northampton, MA, USA). Differences in fatigue characteristics were tested using two-way repeated-measures ANOVA with *post hoc* Tukey tests. Biochemical variables were compared between the three experimental groups using a one-way ANOVA. Statistical significance was accepted when *p* < 0.05. Data are means ± SE.

## 3. Results

No significant differences in glucose or cholesterol concentrations in plasma were observed between control and 1 mg ASTA/kg BW-fed animals after 45 days of supplementation. Although significant 40% increase in triacylglycerol concentration was measured in plasma of ASTA-fed animals, body weight varied equally in both groups (Table 1).

**Table 1:** Glucose, triacylglycerol (TAG), and cholesterol concentrations in plasma and body weight of Wistar rats supplemented for 45 days with 1 mg ASTA/kg body weight (BW). * (*p* < 0.05).

Parameters	Control	ASTA
Glucose (mg/dL)	170.4 ± 33.6	170.6 ± 13.3
TAG (mg/dL)	121.8 ± 17.6	171.4 ± 26.8 (*)
Total cholesterol (mg/dL)	79.1 ± 6.7	82.3 ± 18.7
BW/day 0 (g)	148.4 ± 8.4	158.8 ± 5.9
BW/day 30 (g)	267.5 ± 34.9	299.1 ± 8.0
BW/day 45 (g)	324.2 ± 47.5	347.9 ± 11.0

**Figure 2:**
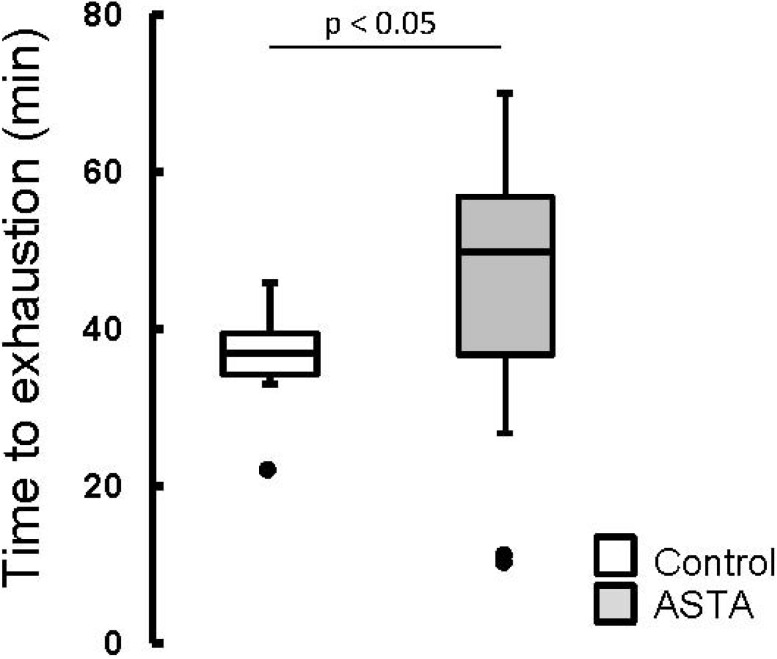
Boxplots of the swimming activity until exhaustion (min) of Wistar rats loaded with additional 5% BW (to force swimming) and supplemented for 45 days with 1 mg ASTA/kg BW or mineral oil (control). (*n* ≥ 11, *p* < 0.05). (•) Outliner data of the IQR = 1.25 × (Q3 − Q1), where: IQR = interquartile range; Q3 = 75% quartile; and Q1 = 25% quartile.

The elapsed time until exhaustion of forced-swimming activity was 29% higher in ASTA-fed animals (48.2 ± 14.1 min) than in control (37.4 ± 4.0 min), as shown by boxplots in Figure 2.

Exercise to exhaustion significantly changed the redox balance in the plasma of animals. As shown in Table 2, uric acid concentrations in plasma of C-EX and ASTA-EX animals increased by 160% in both groups, independently of supplementation. On the other hand, we observed a 2.6-fold higher concentration of iron ions in C-EX compared to C group, whereas iron content in plasma of ASTA-EX animals was only 80% higher than in ASTA-fed animals. The concentration of heme-iron in plasma did not vary with exhaustion under our experimental conditions. Moreover, exhaustion did not significantly decrease TEAC antioxidant activity in plasma of ASTA-fed animals, although a slight tendency to diminish was observed in control group (*p* = 0.0825). Noteworthy, ASTA supplementation itself apparently increased the antioxidant capacity in plasma, as TEAC levels in ASTA and ASTA-EX animals were 22% and 35% higher than in respective C and C-EX groups. Depletion of the antioxidant iron-chelating content in plasma (FRAP activity) after exercise was only confirmed in control group (45% lower), whereas FRAP capacity in ASTA-fed animals increased 62% instead. All these data are summarized in Table 2.

**Table 2:** Oxidative stress biomarkers in plasma of rested or exhausted Wistar rats supplemented for 45 days with 1 mg ASTA/kg BW (ASTA and ASTA-EX, respectively for rested and exhausted animals) or mineral oil (C and C-EX).

Biomarker	C	C-EX	ASTA	ASTA-EX
Uric acid (mg/mL)	0.70 ± 0.04	1.83 ± 0.26 ^‡^ (C,A)	0.66 ± 0.04 ^‡^ (CE,AE)	1.74 ± 0.10 ^‡^ (C,A)
TEAC (nmolEq.Trolox/mL)	1.73 ± 0.10	1.50 ± 0.20	2.11 ± 0.07 * (C,CE)	2.03 ± 0.13 * (CE)
Iron (µg/dL)	8.70 ± 0.72	21.26 ± 4.28 ^†^ (C) * (AE), ^‡^ (A)	7.43 ± 0.69 ^‡^ (CE,AE)	13.33 ± 1.27 * (C,CE), ^‡^ (A)
Heme-iron (µg/mL)	32.8 ± 7.4	30.5 ± 6.0	25.4 ± 5.6	28.2 ± 6.5
FRAP (µmol Fe^2+^/min/mL)	2.16 ± 0.26	1.20 ± 0.23 * (C,AE)	1.57 ± 0.14 * (C,AE)	2.55 ± 0.45 * (CE, A)

* (*p* < 0.05); ^†^ (*p* < 0.01); ^‡^ (*p* < 0.005), compared to: control (C), control exhausted (CE), ASTA-fed (A), or ASTA-fed exhausted animals (AE) (*n* = 6).

**Figure 3:**
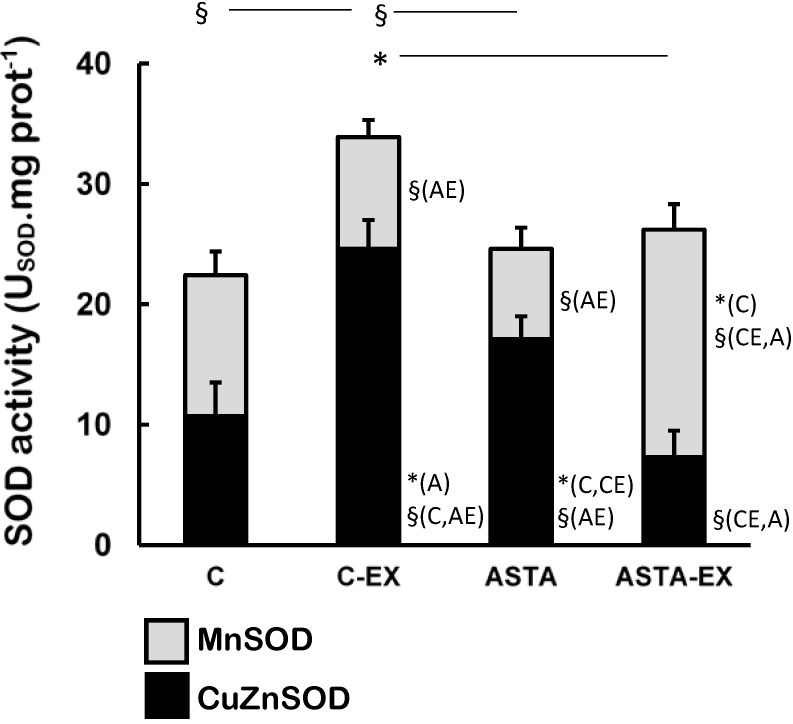
Enzyme activities of total-, CuZn- (cytosolic), and Mn-dependent (mitochondrial) SOD in soleus muscles of Wistar rats submitted to exhaustion (forced swimming activity with additional 5% BW) and supplemented for 45 days with 1 mg ASTA/kg BW (ASTA or ASTA-EX) or mineral oil as control (C or C-EX) (*n* = 6) * (*p* < 0.05); § (*p* < 0.005), compared to: control (C), control exhausted (CE), ASTA-fed (A), or ASTA-fed exhausted (AE).

Exhaustion affected the redox balance in soleus muscles of control animals, as shown by a 55% increase of total SOD activity in C-EX muscles, but not in ASTA-EX (Figure 3). It is worthy to note that total SOD was represented in Figure 3 as the sum of the enzyme activities of the cytosolic CuZnSOD (black bars) and the mitochondrial MnSOD isoforms (light gray bars). No changes were observed in MnSOD activity between C and C-EX groups. On the other hand, a hypothetical compensatory effect between CuZnSOD and MnSOD (favoring the mitochondrial isoform MnSOD) was observed in ASTA-fed animals, which resulted in unaltered total SOD activities upon exhaustion.

**Figure 4:**
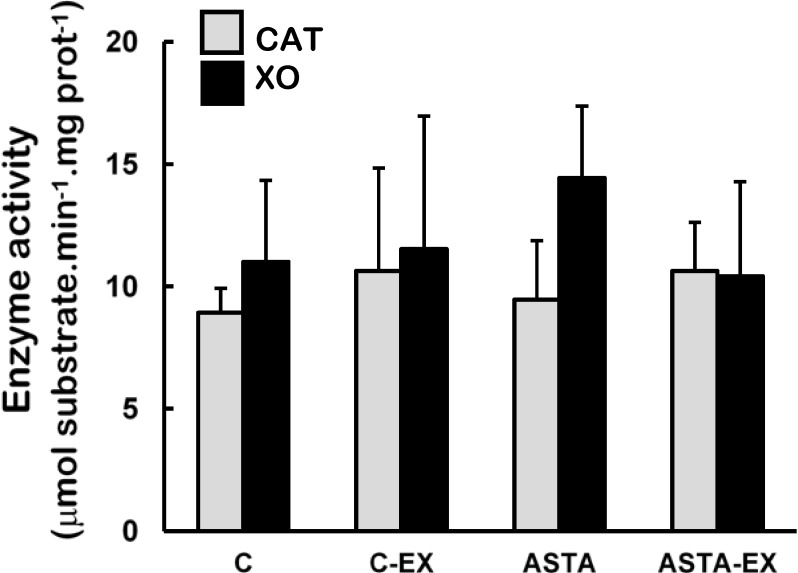
Enzyme activities of catalase (CAT) and xanthine oxidase (XO) in soleus muscles of Wistar rats submitted to exhaustion (forced swimming activity with additional 5% BW) and supplemented for 45 days with 1 mg ASTA/kg BW (ASTA or ASTA-EX) or mineral oil as control (C or C-EX). (*n* = 6).

**Figure 5:**
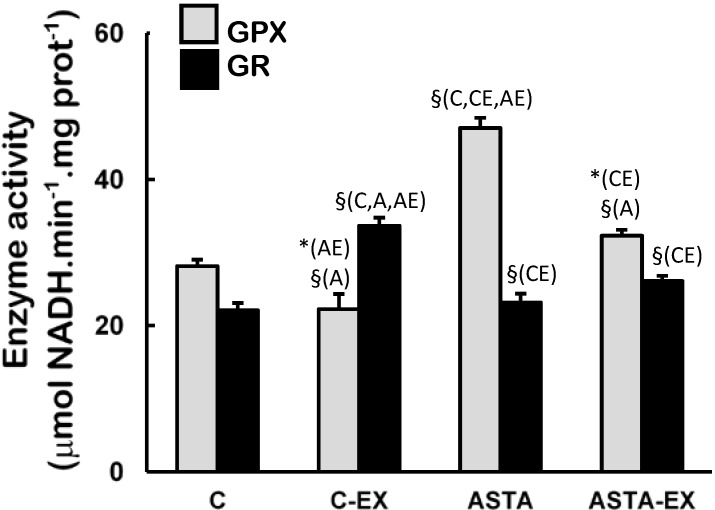
Enzyme activities of glutathione peroxidase (GPX) and glutathione reductase (GR) in soleus muscles of Wistar rats submitted to exhaustion (forced swimming activity with additional 5% BW) and supplemented for 45 days with 1 mg ASTA/kg BW (ASTA or ASTA-EX) or mineral oil as control (C or C-EX). (*n* = 6).* (*p* < 0.05); § (*p* < 0.005), compared to: control (C), control exhausted (CE), ASTA-fed (A), or ASTA-fed exhausted (AE).

Catalase (CAT) and xanthine oxidase (XO) activities were unaltered in soleus muscles of all experimental groups (Figure 4). On the other hand, long-term ASTA supplementation improved basal GPX antioxidant defenses in soleus muscles of ASTA group (versus C group) by approximately 60%, although no difference was observed in GR activity (Figure 5). Exhaustion caused a 60% increase of GR activity only in muscles of control animals. GPX activities in soleus muscles decreased after exercise in both groups, although the decrease in ASTA-fed animals was more pronounced (30% drop).

Finally, Table 3 compiles information of the thiol/glutathione (GSH) contents in soleus muscles of experimental animals: (i) in control, exhaustion caused massive drops in protein thiol and GSSG contents (50% and 55%, respectively), but a 26% increase in GSH content; and (ii) in ASTA-fed animals, although similar decrease of GSSG content was observed (50%), both GSH and protein thiol contents were increased after exhaustion (88% and 53%, respectively). Based on the GSH-dependent reducing power, lower increments in GSH/GSSG ratio was necessary in ASTA-EX than in C-Ex group.

**Figure 6:**
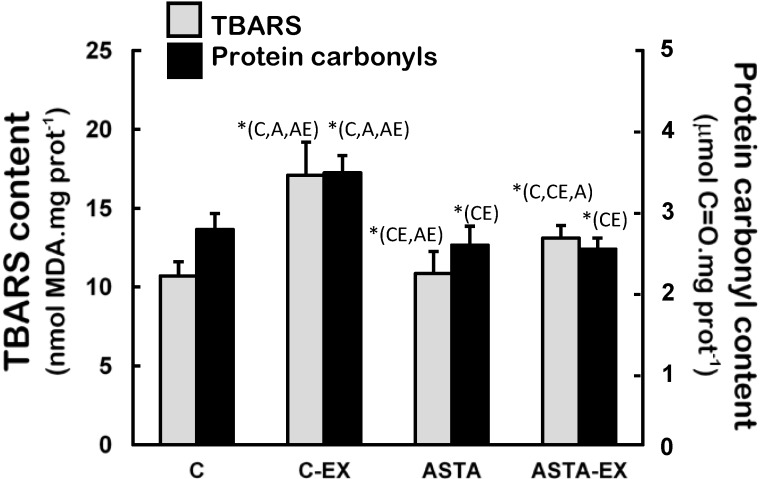
Concentration of biomarkers of oxidative modifications in lipids (TBARS assay) and proteins (protein carbonyls) in soleus muscles of Wistar rats submitted to exhaustion (forced swimming activity with additional 5% BW) and supplemented for 45 days with 1 mg ASTA/kg BW or mineral oil (control). (*n* = 6).* (*p* < 0.05), compared to: control (C), control exhausted (CE), ASTA-fed (A), or ASTA-fed exhausted (AE).

Figure 6 demonstrates that soleus muscles of C-EX group were highly exposed to oxidative conditions, since levels of oxidized lipids (60% higher TBARS levels) and proteins (33% higher protein carbonyls) were significantly different to control. Milder increments of lipid oxidation products were measured in ASTA-EX animals (28% higher than ASTA), but no changes in protein oxidation were evidenced.

**Table 3:** Concentration of reduced (GSH), oxidized glutathione (GSSG), their ratio (GSH/GSSG), and protein thiol content in soleus muscles of Wistar rats submitted to exhaustion (forced swimming activity with additional 5% BW) and supplemented for 45 days with 1 mg ASTA/kg BW or mineral oil (control) (*n* = 6).

Parameters	C	C-EX	ASTA	ASTA-EX
GSH (nmol/g tissue)	51.26 ± 0.91	64.54 ± 9.30 *(C), ^‡^ (A), ^†^ (AE)	19.72 ± 3.18 ^‡^ (C,CE,AE)	37.14 ± 3.99 * (C), ^†^ (CE), ^‡^ (A)
GSSG (nmol/g tissue)	220.0 ± 4.0	98.3 ± 31.2 ^†^ (C)	183.1 ± 33.5 * (AE)	94.0 ± 14.0 ^‡^ (C), * (A)
Protein thiols (µmol-SH/mg prot)	4.48 ± 1.62	2.31 ± 0.34 * (C)	3.76 ± 0.17 * (CE)	5.77 ± 2.88
Ratio GSH/GSSG (U.A.)	0.233 ± 0.006	0.656 ± 0.229 * (C), ^‡^ (A)	0.108 ± 0.026 * (C,AE), ^‡^ (CE)	0.395 ± 0.072 * (C,A)

* (*p* < 0.05); ^†^ (*p* < 0.01); ^‡^ (*p* < 0.005), compared to: control (C), control exhausted (CE), ASTA-fed (A), or ASTA-fed exhausted animals (AE).

## 4. Discussion

We have found that long-term ASTA supplementation in Wistar rats significantly delayed exhaustion in a forced-swimming test. Adequate redox balance in plasma and especially in soleus muscles were associated with the ameliorated performance.

ASTA is apparently well absorbed by the gastroenteric system of mammals. In plasma, ASTA is biotransformed by several liver CYP-like enzymes and converted into β-ionone derivatives [28]. Based on those principles, we opted for a long-term supplementation protocol (45 days, 5 days/week) aiming the maximal accumulation of the lipid-soluble carotenoid ASTA in rat tissues. Scarce information is currently available regarding ASTA accumulation in rat/human muscles [29]. Low concentrations of ASTA were previously detected in most of the internal organs (spleen > adrenals > kidneys >> liver ~ eyes), but especially in the skin of rats [30]. In salmon, for example, if typical values for the dietary ASTA concentration (50 mg/kg) and feed conversion ratio (FCR 1.15) are used, 26% of the absorbed ASTA is retained by muscles [31]. Although carotenoid concentrations were not measured in rat plasma or muscles here, we studied ASTA antioxidant effects at a systemic (plasma) and local levels (in soleus muscles) by measuring key metabolites and enzyme activities in both biological matrices. ASTA supplementation did not alter glycemia, cholesterolemia or body weight (BW) in Wistar animals compared to controls, although plasma triacylglycerol content (TAG) was significantly increased (Table 1).

Exhaustion is described as a condition when muscles are unable to perform due to inefficient ATP supply for the contractile activity, either caused by insufficient fuel provision (fatty acid or glucose/glycogen oxidation) or other related events, such as the free radical-mediated dysfunction of the ATP-generating system [14,32]. Exhausted muscles display an unfavorable AMP/ATP ratio that stimulates the purine catabolic pathway, through xanthine oxidase (XO), leading to uric acid accumulation in tissues and plasma [33,34]. Superoxide radical and hydrogen peroxide (O_2_^•−^ and H_2_O_2_, respectively) are concomitantly produced during the process, which enhance exercise-related oxidative stress. Although XO activities were unaltered in exhausted muscles of animals here (Figure 4), both control and ASTA-fed groups showed identical 2.6-fold higher uric acid (UA) concentrations in plasma after exercise, suggesting similar physical efforts from all animals to reach exhaustion (Table 2). Nevertheless, many tissues/cells could contribute for the detected XO activity in plasma, e.g., skeletal muscles, vascular endothelium, *etc.*, which could explain the unaltered XO activities measured in plasma despite significant variations in UA were concomitantly measured (Figure 4 and Table 2).

ASTA supplementation clearly delayed exhaustion by 29%, considering the total time of swimming activity (Figure 2). Accordingly, ASTA supplementation (4 mg/day, for 28 days) also improved performance in professional cyclists, although authors did not elucidate the mechanism involved [35]. However, controversial results (no positive effects on performance or some redox indexes in plasma) were observed when ASTA was also administered at higher amounts to well-trained cyclists (20 mg/day) [36]. Nevertheless, Table 2 also suggests that ASTA supplementation limited the progression of oxidative events in plasma imposed by exhaustive exercise. In control, 2.6-fold higher concentrations of iron ions in plasma (C-EX *versus* C, Table 2) were not counterbalanced by proportional increases of iron-chelating/scavenging capacity in plasma (FRAP/TEAC activities). On the other hand, ASTA supplementation induced metabolic/physiological adaptations that resulted in lower iron overload in plasma following exercise (ASTA-EX 80% higher than ASTA, Table 2). ASTA-EX animals also showed incremented FRAP/TEAC defenses to cope with the pro-oxidant conditions imposed by iron overload in plasma (Table 2). It is worthy to note that ASTA supplementation had already increased background TEAC activity in plasma by 22% (ASTA group versus C, Table 2), in parallel to a slight tendency for lower iron contents (*p* = 0.0772). Accordingly, long-term ASTA supplementation of trained young soccer players (90 days) also limited antioxidant depletion in plasma and decreased indexes of muscle damage after a 2-h acute exercise bout [37]. Although ASTA supplementation altered iron homeostasis during exercise (Table 2), heme-iron content did not vary significantly, a fact that weakens the putative contribution of rhabdomyolysis or hemolysis (related to, respectively, myoglobin and hemoglobin release in plasma) on pre/post-exercise iron overload [38].

Antioxidant systems—low molecular weight compounds, proteins and enzymes—undergo adaptive modulation in response to short- and long-term exercise, in an attempt to sustain an adequate redox balance in working muscles [39]. Under our experimental conditions here, exhaustion was clearly associated to oxidative stress in soleus muscles of control animals: significant increases in total SOD activities after exercise (mainly from cytosolic sources, as suggested by changes in CuZnSOD activities in Figure 3), but not proportional responses from key H_2_O_2_-removing enzymes CAT and GPX (Figure 4 and Figure 5, respectively). Adaptive increases in GSH/GSSG ratios due to activation of GR activity match the harmful oxidative insult imposed by exhaustive exercise to C-EX animals (Table 3 and Figure 5). However, none of the observed antioxidant responses was sufficient to avoid oxidative stress in working muscles, as demonstrated by higher concentrations oxidized lipids (TBARS levels, Figure 5) and proteins (carbonyls and thiols, respectively in Figure 5 and Table 2). The hypothesis of exacerbated oxidative stress in soleus muscles of C-EX animals is corroborated by plasma results (Table 1).

Interestingly, soleus muscles of ASTA-EX animals apparently displayed more adequate antioxidant responses to cope with the exercise-induced oxidative stress. Aoi *et al*. (2008) elegantly showed that ASTA treatment also increased time to exhaustion in treadmill tests by improving the utilization of lipids as energy substrates during exercise [40]. However, the authors left some questions regarding the redox imbalances provoked by ROS/RNS overproduction during exercise. In agreement to Aoi *et al*. 2008 observations, we also observed hyperlipidemic effects of ASTA here (TAG in Table 1) and in our previous studies [41]. Our results are in agreement with the hypothesis that ASTA supplementation induces higher consumption of lipids as fuels for exercise, with higher mitochondrial demands through β-oxidation in working muscles and proportional increase of ROS/RNS production [42,43]. Therefore, it is not surprising that the mitochondrial isoform MnSOD was induced in ASTA-EX muscles, whereas CuZnSOD was diminished (Figure 3). Further studies are necessary to understand how ASTA apparently induces hyperlipidemia and possibly increases the mitochondrial production of ROS/RNS in exercising muscles.

In fact, several studies demonstrated that ASTA putatively alters mitochondrial metabolism: (i) ASTA shows antioxidant and anti-apoptotic effects in MPP^+^-induced culture cells via induction of expression of superoxide dismutase (SOD) and regulation of Bcl-2 and Bax expression [44]; (ii) ASTA increased the co-localization of fatty-acid translocase (FAT/CD36) with carnitine-palmitoyltransferase I (CPT I) and prevented the oxidative modification of CPT I in exercise [40]; (iii) ASTA pretreatment significantly inhibited apoptosis, mitochondrial abnormalities and intracellular ROS generation observed in 6-hydroxydopamine-treated SH-SY5Y cells [45]; and (iv) ASTA treatment improved mitochondrial function of leukocytes in young and, especially, aged dogs by increasing ATP synthesis, mitochondria mass, and cytochrome c oxidoreductase activity [46]. Regarding prolonged (aerobic-like) exercise, the mitochondrial-targeted action of ASTA is particularly relevant, since soleus muscles are mostly composed by slow-twitch/oxidative type I fibers, provided with high mitochondrial populations [47]. Accordingly, Liu *et al*. (2014) have recently demonstrated that exercised ASTA-fed mice enhance lipid consumption as fuel for the contractile activity, with less contribution from glucose oxidation. As stated by the authors: “Because energy consumed in muscle during exercise is mainly supplied by carbohydrates and lipids, astaxanthin-induced lipid utilization can decrease energy obtained from carbohydrates, which may lead to the decrease in lactate/proton production” [48]. The mechanism possibly involves mitochondrial activation, since peroxisome proliferator-activated receptor-γ coactivator-1α (PGC-1α) and its downstream proteins were concomitantly induced [48]. An identical interpretation—based on ASTA effects on mitochondria—could explain the controversial results in resistance-trained men indicating that ASTA did not favorably affect indirect markers of skeletal muscle injury following anaerobic-like eccentric loading [49].

ASTA supplementation provided extended protection to lipids and proteins in soleus muscles of rats during exercise, which could be associated to prolonged swimming activity before exhaustion (Figure 2). ASTA is well defined as an efficient scavenger of ROO• and RO• radicals, which are very reactive promoters of lipid peroxidation [50]. Relatively stable organic peroxides (ROOH) are final products of lipid peroxidation, although ROO•/RO• radicals could be regenerated from ROOH in the presence of redox-active iron ions [51]. GPX is a key enzyme in redox metabolism since it catalyzes the decomposition of the potentially harmful peroxides ROOH and H_2_O_2_, and directly affects GSH concentration in biological systems [52]. At rest, the higher activity of GPX in soleus muscles of ASTA-fed animals was responsible for the efficient removal of ROOH and H_2_O_2_ and, thereby, limited protein and lipid oxidation (Figure 4 and Figure 5). Data suggest that iron metabolism was under control at rest (based on plasma levels, Table 2). Moreover, low concentrations of GSH (and GSH/GSSG ratios) were accordingly measured in rested soleus muscles, because GSH is the major substrate of cytosolic GPX. On the other hand, the induction of MnSOD in soleus muscles of ASTA-EX animals suggests that exhaustive exercise increased ROS/RNS in mitochondria. The hypothetical mitochondrial-centered action of ASTA would, therefore, scavenge ROO•/RO• radicals produced in mitochondria-rich fibers of soleus muscles and limit ROOH production during exhaustive exercise. Apparently, this is a crucial event to limit lipid/protein oxidation in soleus muscles, since iron homeostasis is harmfully disrupted during exhaustive exercise (Table 2). Lower loads of ROOH would allow GPX to prioritize the removal of increasing amounts of H_2_O_2_ produced by induced (mitochondrial) MnSOD dismutation in exercising muscles. In agreement, lower GPX activities match the substantial increase in GSH concentration (and GSH/GSSG ratio) in exhausted soleus muscles: (i) C-EX showed 26% higher GSH content and 2.8-fold higher GSH/GSSG ratio compared to C group, whereas ASTA-EX showed 88% and 3.7-fold increments (compared to ASTA), respectively. Considering that GSH is also an efficient free radical scavenger in biological systems [53], the ASTA-induced decrease in GPX activity in soleus muscles is here assumed as an additional metabolic adaptation to supply GSH units in order to sustain the positive redox balance during the swimming test.

One of the most accepted theories to explain the physiological benefits provided by regular exercise refers to a retrograde response called hormesis. Hormesis represents the result of the moderate prevalence of higher mitochondrial/cellular ROS formation over the antioxidant adaptations, which culminates in an upgraded stress resistance (acquired muscle antioxidant adaptations and redox-sensitive gene expression) and controlled accumulation of oxidative damage within the intracellular environment. Hence, the hormesis mechanism could sustain longer periods of optimized mitochondrial/cellular functions due to a positive ATP-supply/oxidative damage ratio within cells [54]. Therefore, based on the hormesis principle, it is possible that excessive antioxidant activity in cells could hinder the essential redox-mediated responses, necessary to obtain the physiological benefits from regular (and moderate) exercise [51,52]. This is a subject of major concern when antioxidant supplementation is under discussion.

## 5. Conclusions

Long-term ASTA supplementation in Wistar rats significantly delayed exhaustion in a forced-swimming test. Adequate redox balance in plasma and especially in soleus muscles were associated to the ameliorated performance. Possible mechanisms by which ASTA limited oxidative stress in plasma and muscle tissues, include: (i) ASTA increased scavenging/iron-chelating (TEAC/FRAP) capacities in plasma limiting iron overload and its related pro-oxidant effects; (ii) ASTA induced significant mitochondrial antioxidant responses (MnSOD) that apparently sustained ATP generation in mitochondria-rich soleus muscles; (iii) ASTA directly scavenged ROO•/RO• radicals, key promoters of lipid peroxidation; (iv) by damping down ROOH production, ASTA indirectly favored the removal of mitochondrial-produced H_2_O_2_ by GPX in soleus muscles; and (v) by inducing lower GPX activities, ASTA indirectly incremented the intracellular antioxidant defense, by enhancing GSH levels and GSH/GSSG ratios in ASTA-EX muscles.

As shown by other authors, “mitochondrial nutrients” can reduce oxidative stress and mitochondrial dysfunction in many (patho)physiological conditions mediated by ROS/RNS [55,56]. On a larger scale, ASTA supplementation can be suggested as a nutritional additive to improve aerobic-like exercise performance in humans, with other putative health benefits associated, especially against ROS/RNS-related diseases [57].

## References

[1] Palozza P., Krinsky N.I. (1992). Astaxanthin and canthaxanthin are potent antioxidants in a membrane model. Arch. Biochem. Biophys..

[2] Wolf A.M., Asoh S., Hiranuma H., Ohsawa I., Iio K., Satou A., Ishikura M., Ohta S. (2010). Astaxanthin protects mitochondrial redox state and functional integrity against oxidative stress. J. Nutr. Biochem..

[3] Guerin M., Huntley M.E., Olaizola M. (2003). *Haematococcus* astaxanthin: Applications for human health and nutrition. Trends Biotechnol..

[4] Urso M.L., Clarkson P.M. (2003). Oxidative stress, exercise, and antioxidant supplementation. Toxicology.

[5] Cooper C.E., Vollaard N.B., Choueiri T., Wilson M.T. (2002). Exercise, free radicals and oxidative stress. Biochem. Soc. Trans..

[6] Radak Z., Zhao Z., Koltai E., Ohno H., Atalay M. (2013). Oxygen consumption and usage during physical exercise: The balance between oxidative stress and ROS-dependent adaptive signaling. Antioxid. Redox Signal..

[7] Xu J., Hwang J.C.Y., Lees H.A., Wohlgemuth S.E., Knutson M.D., Judge A.R., Dupont-Versteegden E.E., Marzetti E., Leeuwenburgh C. (2012). Long-term perturbation of muscle iron homeostasis following hindlimb suspension in old rats is associated with high levels of oxidative stress and impaired recovery from atrophy. Exp. Gerontol..

[8] Barros M.P., Ganini D., Lorenço-Lima L., Soares C.O., Pereira B., Bechara E.J.H., Silveira L.R., Curi R., Souza-Junior T.P. (2011). Effects of acute creatine supplementation on iron homeostasis and uric acid-based antioxidant capacity of plasma after wingate test. J. Int. Soc. Sports Nutr..

[9] Ibrahim M.Y., Ashour O.M. (2011). Changes in nitric oxide and free radical levels in rat gastrocnemius muscle during contraction and fatigue. Clin. Exp. Pharmacol. Physiol..

[10] Kobayashi M., Kurimura Y., Sakamoto Y., Tsuji Y. (1997). Selective extraction of astaxanthin and chlorophyll from the green alga *Haematococcus pluvialis*. Biotechnol. Tech..

[11] Stewart J.S., Lignell A., Pettersson A., Elfving E., Soni M.G. (2008). Safety assessment of astaxanthin-rich microalgae biomass: Acute and subchronic toxicity studies in rats. Food Chem. Toxicol..

[12] Pereira B., Costa-Rosa L.F.B.P., Safi D.A., Guimarães A.R.P., Bechara E.J.H., Curi R. (1994). Antioxidant enzyme activities in the lymphoid organs and muscles of rats fed fatty Acids-rich diets subjected to prolonged physical exercise-training. Physiol. Behav..

[13] Lancha A.H., Recco M.B., Abdalla D.S.P., Curi R. (1995). Effect of aspartate, asparagine and carnitine supplementation in the diet on metabolism of skeletal muscle during a moderate exercise. Physiol. Behav..

[14] Finsterer J. (2012). Biomarkers of peripheral muscle fatigue during exercise. BMC Musculoskelet. Disord..

[15] Van den Berg R., Haenen G.R.M.M., Van den Berg H., Bast A. (1999). Applicability of an improved Trolox equivalent antioxidant capacity (TEAC) assay for evaluation of antioxidant capacity measurements of mixtures. Food Chem..

[16] Benzie I.F.F., Strain J.J. (1996). The Ferric Reducing Ability of Plasma (FRAP) as a measure of ‘‘Antioxidant Power’’: The FRAP Assay. Anal. Biochem..

[17] Brewer K.J., Murphy J.W.R., Petersen J.D. (1987). Synthesis and characterization of monometallic and bimetallic mixed-ligand complexes of iron (II) containing 2,2′-bipyrimidine or 2,3-bis(2-pyridyl) pyrazine. Inorg. Chem..

[18] Van Kampen E.J., Zijlstra W.G. (1965). Determination of hemoglobin and its derivatives. Adv. Clin. Chem..

[19] Ewing J.F., Janero D.R. (1995). Microplate superoxide dismutase assay employing a nonenzymatic superoxide generator. Anal. Biochem..

[20] Aebi H. (1984). Catalase *in vitro*. Methods Enzymol..

[21] Mannervik B. (1985). Glutathione peroxidase. Methods Enzymol..

[22] Cunningham S.K., Keaveny T.V. (1978). A two-stage enzymatic method for determination of uric acid and hypoxanthine/xanthine. Clin. Chim. Acta.

[23] Levine R.L., Williams J.A., Stadtman E.R., Shacter E. (1994). Carbonyl assays for determination of oxidatively modified proteins. Methods Enzymol..

[24] Akerboom T.P.M., Sies H. (1981). Assay of glutathione, glutathione disulfide, and glutathione mixed disulfides in biological samples. Methods Enzymol..

[25] Fraga C.G., Leibovitz B.E., Tappel A.L. (1988). Lipid peroxidation measured as thiobarbituric acid-reactive substances in tissue slices: Characterization and comparison with homogenates and microsomes. Free Radic. Biol. Med..

[26] Rose R.C., Bode A.M. (1995). Analysis of water-soluble antioxidants by high pressure liquid chromatography. Biochem. J..

[27] Rodriguez-Ariza A., Toribio F., López-Barea J. (1994). Rapid determination of glutathione status in fish liver using high-performance liquid chromatography and electrochemical detection. J. Chromatogr. B Biomed. Appl..

[28] Kistler A., Liechti H., Pichard L., Wolz E., Oesterhelt G., Hayes A., Maurel P. (2002). Metabolism and CYP-inducer properties of astaxanthin in man and primary human hepatocytes. Arch. Toxicol..

[29] Aoi W., Naito Y., Sakuma K., Kuchide M., Tokuda H., Maoka T., Toyokuni S., Oka S., Yasuhara M., Yoshikawa T. (2003). Astaxanthin limits exercise-induced skeletal and cardiac muscle damage in mice. Antioxid. Redox Signal..

[30] Petri D., Lundebye A.K. (2007). Tissue distribution of astaxanthin in rats following exposure to graded levels in the feed. Comp. Biochem. Physiol. C.

[31] Ytrestøyl T., Bjerkeng B. (2007). Intraperitoneal and dietary administration of astaxanthin in rainbow trout (*Oncorhynchus mykiss*)—Plasma uptake and tissue distribution of geometrical E/Z isomers. Comp. Biochem. Physiol. B.

[32] Westerblad H., Allen D.G. (2011). Emerging roles of ROS/RNS in muscle function and fatigue. Antioxid. Redox Signal..

[33] Souza-Junior T.P., Lorenço-Lima L., Ganini D., Vardaris C.V., Polotow T.G., Barros M.P. (2014). Delayed uric acid accumulation in plasma provides additional antioxidant protection against iron-triggered oxidative stress after a Wingate test. Biol. Sport.

[34] Sachdev S., Davies K.J. (2008). Production, detection, and adaptive responses to free radicals in exercise. Free Radic. Biol. Med..

[35] Earnest C.P., Lupo M., White K.M., Church T.S. (2011). Effect of astaxanthin on cycling time trial performance. Int. J. Sports Med..

[36] Res P.T., Cermak N.M., Stinkens R., Tollakson T.J., Haenen G.R., Bast A., Van Loon L.J.C. (2013). Astaxanthin supplementation does not augment fat use or improve endurance performance. Med. Sci. Sports Exerc..

[37] Djordjevic B., Baralic I., Kotur-Stevuljevic J., Stefanovic A., Ivanisevic J., Radivojevic N., Andjelkovic M., Dikic N. (2012). Effect of astaxanthin supplementation on muscle damage and oxidative stress markers in elite young soccer players. J. Sports Med. Phys. Fitness.

[38] Kratz A., Lewandrowski K.B., Siegel A.J., Chun K.Y., Flood J.G., Van Cott E.M., Lee-Lewandrowski E. (2002). Effect of marathon running on hematologic and biochemical laboratory parameters, including cardiac markers. Am. J. Clin. Pathol..

[39] Aguiló A., Tauler P., Fuentespina E., Tur J.A., Córdova A., Pons A. (2005). Antioxidant response to oxidative stress induced by exhaustive exercise. Physiol. Behav..

[40] Aoi W., Naito Y., Takanami Y., Ishii T., Kawai Y., Akagiri S., Kato Y., Osawa T., Yoshikawa T. (2008). Astaxanthin improves muscle lipid metabolism in exercise via inhibitory effect of oxidative CPT I modification. Biochem. Biophys. Res. Commun..

[41] Barros M.P., Marin D.P., Bolin A.P., Macedo R.C.S., Campoio T.R., Fineto Jr. C., Guerra B.A., Polotow T.G., Vardaris C.V., Mattei R. (2012). Combined astaxanthin and fish oil supplementation improves glutathione-based redox balance in rat plasma and neutrophils. Chem. Biol. Interact..

[42] Ikeuchi M., Koyama T., Takahashi J., Yazawa K. (2006). Effects of astaxanthin supplementation on exercise-induced fatigue in mice. Biol. Pharm. Bull..

[43] Schrauwen P., Hesselink M.K. (2004). Oxidative capacity, lipotoxicity, and mitochondrial damage in type 2 diabetes. Diabetes.

[44] Lee D.H., Kim C.S., Lee Y.J. (2011). Astaxanthin protects against MPTP/MPP+-induced mitochondrial dysfunction and ROS production *in vivo* and *in vitro*. Food Chem. Toxicol..

[45] Liu X., Shibata T., Hisaka S., Osawa T. (2009). Astaxanthin inhibits reactive oxygen species-mediated cellular toxicity in dopaminergic SH-SY5Y cells via mitochondria-targeted protective mechanism. Brain Res..

[46] Park J.S., Mathison B.D., Hayek M.G., Zhang J., Reinhart G.A., Chew B.P. (2013). Astaxanthin modulates age-associated mitochondrial dysfunction in healthy dogs. J. Animal Sci..

[47] Fitts R.H. (1994). Cellular mechanisms of muscle fatigue. Physiol. Rev..

[48] Liu P.H., Aoi W., Takami M., Terajima H., Tanimura Y., Naito Y., Itoh Y., Yoshikawa T. (2014). The astaxanthin-induced improvement in lipid metabolism during exercise is mediated by a PGC-1α increase in skeletal muscle. J. Clin. Biochem. Nutr..

[49] Bloomer R.J., Fry A., Schilling B., Chiu L., Hori N., Weiss L. (2005). Astaxanthin supplementation does not attenuate muscle injury following eccentric exercise in resistance-trained men. Int. J. Sport Nutr. Exerc. Metab..

[50] Barros M.P., Pinto E., Colepicolo P., Pedersén M. (2001). Astaxanthin and peridinin inhibit oxidative damage in Fe (2+)-loaded liposomes: Scavenging oxyradicals or changing membrane permeability?. Biochem. Biophys. Res. Commun..

[51] Stolze K., Udilova N., Nohl H. (2000). Spin trapping of lipid radicals with DEPMPO-derived spin traps: detection of superoxide, alkyl and alkoxyl radicals in aqueous and lipid phase. Free Radic. Biol. Med..

[52] Ji L.L. (2008). Modulation of skeletal muscle antioxidant defense by exercise: Role of redox signaling. Free Radic. Biol. Med..

[53] Masella R., Di Benedetto R., Vari R., Filesi C., Giovannini C. (2005). Novel mechanisms of natural antioxidant compounds in biological systems: Involvement of glutathione and glutathione-related enzymes. J. Nutr. Biochem..

[54] Ristow M., Zarse K. (2010). How increased oxidative stress promotes longevity and metabolic health: The concept of mitochondrial hormesis (mitohormesis). Exp. Gerontol..

[55] Oyewole A.O., Wilmot M.C., Fowler M., Birch-Machin M.A. (2014). Comparing the effects of mitochondrial targeted and localized antioxidants with cellular antioxidants in human skin cells exposed to UVA and hydrogen peroxide. FASEB J..

[56] Mercer J.R., Yu E., Figg N., Cheng K.K., Prime T.A., Griffin J.L., Masoodi M., Vidal-Puig A., Murphy M.P., Bennett M.R. (2012). The mitochondria-targeted antioxidant MitoQ decreases features of the metabolic syndrome in ATM+/−/ApoE−/− mice. Free Radic. Biol. Med..

[57] Barros M.P., Poppe S.C., Souza-Junior T.P. (2011). Putative benefits of microalgal astaxanthin on exercise and human health. Braz. J. Pharm..

